# Nonlinear Acoustic Modeling and Measurements during the Fatigue Process in Metals

**DOI:** 10.3390/ma12040607

**Published:** 2019-02-18

**Authors:** Wenhan Lyu, Xianmei Wu, Weijiang Xu

**Affiliations:** 1 State Key Laboratory of Acoustics, Institute of Acoustics, Chinese Academy of Sciences, Beijing 100190, China; lvwenhan@mail.ioa.ac.cn; 2 University of Chinese Academy of Sciences, Beijing 100049, China; 3 Université Polytechnique Hauts-de-France, CNRS, Univ. Lille, YNCREA, Centrale Lille, UMR 8520IEMN-DOAE, F-59313 Valenciennes CEDEX 9, France; wei-jiang.xu@uphf.fr

**Keywords:** nonlinear spring model, dislocation dipoles, fatigue evaluation

## Abstract

The nonlinear spring model combined with dislocation dipole theory was applied to describe the acoustic nonlinearity during the fatigue process in metals. The spring stiffness changes with fatigue degree. For the early stage, spring stiffness approaches infinity, and the heavier nonlinearity mainly results from the increase of dislocation density. Further fatigue leads to the occurrence of micro-cracks, during which spring stiffness begins to decrease. Abundant micro-crack sprouting accelerates the crack’s expansion, and spring stiffness drops quickly, which causes the obvious decline in the transmitted harmonic amplitudes. Solutions obtained from the nonlinear wave equation with dislocation terms were added into the spring model. Varying spring stiffness was chosen for simulating the fatigue process. Then, nonlinear harmonic variation during this process was observed, which was classified into three stages: (I) the early dislocation fatigue stage; (II) the micro-crack sprouting stage; (III) the crack expansion stage. Nonlinear acoustic measurements were carried out on an aluminum alloy specimen during its fatigue process until cracks could be seen clearly. Harmonic variations in experiments can also be classified into the same three stages as the numerical results, which provides a theoretical and experimental reference for fatigue evaluation in metals using the nonlinear acoustic method.

## 1. Introduction

Metal materials are widely used in practical engineering applications, but long-term in-service work makes them prone to failure due to fatigue damages. The linear acoustic method is invalid for detecting fatigue damages of materials, while the finite amplitude sound wave can produce obvious waveform distortion when it propagates through a medium with fatigue damages, which has been proven to be an effective method for evaluating fatigue damages in metal materials [1,2,3,4,5]. 

Nonlinear acoustic theory in solids established by researchers such as Landau, Murnaghan, and Goldberg provides the basis for nonlinear ultrasonic testing [6,7]. Based on this, theoretical models were proposed to explain the mechanism of the nonlinear acoustic effect caused by micro-damages. For early stage fatigue damages are usually described by the dislocation string [8] or dislocation dipole models [9,10], while these are not suitable for heavier damages owing to the sprouting of micro-cracks. The PM (proposed by Preisach and Mayergoyz) model [11] and contact interface model [12,13,14,15] were proposed to explain the nonlinear acoustic mechanism caused by micro-cracks or imperfect interfaces, through which nonlinear effects such as harmonics, difference frequency waves, resonance frequency drift, and slow dynamics can be simulated. Most of the research works have been based on the above theoretical models. For example, reflection and transmission theories at an open or closed interface were established to study the contact nonlinearity of acoustics based on the contact interface model [16,17]. Some experiments [9,10,18,19] were carried out to observe the nonlinear properties of material delamination and microstructure changes caused by fatigue damages, which established the connection between micro-damages and acoustical nonlinearity. Nonlinear Lamb waves have been commonly applied to evaluate the fatigue damages or detect the bonding quality of solid boards [20,21]. Furthermore, some researchers [22] proved the effectiveness of the nonlinear acoustic method in estimating the strength degradation caused by initial damage of concrete materials. High temperature also leads to micro-damages, which can be measured through the ultrasonic nonlinear parameters to evaluate the microstructure changes during the high-temperature degradation process [23]. The spring model is usually used to study the characteristics of nonlinear acoustic waves at the solid rough contact interface to evaluate the interface boundary conditions [14].

Nonlinear acoustics are used in many research works for the non-destructive evaluation of metal materials, but the above research works are mainly focused on one of the stages during the fatigue process, such as early stage fatigue evaluation or crack detection. Actually, fatigue is a continuously changing process, so modeling the whole process is essential for its evaluation. In our work, the nonlinear spring model combined with dislocation dipoles theory was established for modeling the continuous fatigue process from the early dislocation stage to the crack expansion stage. Solutions obtained from the nonlinear wave equation with dislocations [24,25,26,27] were added into the nonlinear spring model. For the early stage, spring stiffness approaches infinity. Fatigue damage is mainly caused by dislocations in this stage. For the late stage damages, varying stiffness was chosen for modeling the different fatigue degree with the sprouting of micro-cracks. Once the crack begins to expand, spring stiffness declines quickly, and the amplitude of each order harmonic drops obviously. The nonlinear acoustic experimental system was built, and measurements were carried out during the whole fatigue process of the sample, which certified the numerical models.

## 2. Theoretical Models

### 2.1. Nonlinearity Due to Lattice Dislocations at Early Stage Fatigue

**Figure 1:**
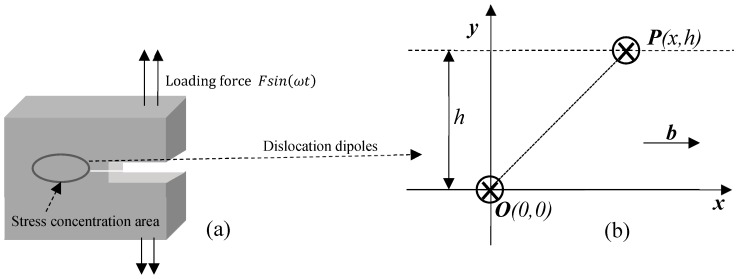
The dislocation dipole model: (**a**) CT specimen (**b**) dislocation dipole.

Early stage fatigue damage of metals mainly originates from dislocations. As the degree of fatigue further deepens, the dislocation density increases progressively, and the nonlinear acoustic effects becomes more obvious. The dislocation can be described by the dipole model [10], as shown in Figure 1.

A compact tension (CT) specimen (Figure 1a) was chosen for modeling. Stress during the fatigue loading mainly concentrates on the initial grooving. At the early stage of the loading process, the appearance of dislocations is considered as pairs of dipoles, as sketched in Figure 1b: (x,h) is the position of one dislocation P in the pair relative to another one O at (0,0). The two dislocation lines are parallel and lie perpendicular to the “*xy*” plane. The unit length glide force [9,10] that acts between the two dislocations in the *x* direction is given as follows:

(1) $$F_{x}=-\frac{Gb^{2}}{2\pi \left(1-v\right)}\frac{x\left(x^{2}-h^{2}\right)}{{\left(x^{2}+h^{2}\right)}^{2}}$$

where *G* is the shear modulus, v is Poisson’s ratio, *b* is the magnitude of Burger’s vector, and *h* represents the spacing between the two glide planes. Propagation of an ultrasonic wave in the material produces the strains of two distinguished origins: the elastic strain $\varepsilon_{l}$ due to the lattice structure of the material and the strain $\varepsilon_{d}$ associated with the motion of dislocation dipoles.

(2) $$\varepsilon =\varepsilon_{l}+\varepsilon_{d}$$

The relationship between the elastic strain $\varepsilon_{l}$ and stress σ is given by [9,28]:

(3) $$\varepsilon_{l}=\frac{1}{A_{2}^{l}}\sigma -\frac{1}{2}\frac{A_{3}^{l}}{{\left(A_{2}^{l}\right)}^{3}}\sigma^{2}+\cdots$$

where $A_{2}^{l}$,$\;A_{3}^{l}$ are the second- and third-order elastic constants, respectively, while the contribution of strain $\varepsilon_{d}$ to the stress σ can be obtained from the dislocation motion equation [10]. Only the relative motion of the dipoles along the direction parallel to the glide planes is considered:

(4) $$m_{eff}\frac{\partial^{2}x}{\partial t^{2}}=F_{x}+b\sigma R$$

where $m_{eff}$ is the effective mass of the dislocation dipoles, and *R* is the Schmid factor. Let ζ=x−h, which is the relative displacement of dislocation dipoles to the position *h*. Combining Equations (1) and (4) and expanding Equation (1) in the series method with ζ , the following is obtained:

(5) $$m_{eff}\frac{\partial^{2}\zeta }{\partial t^{2}}+\frac{Gb^{2}}{4\pi h^{2}\left(1-v\right)}\zeta -\frac{Gb^{2}}{8\pi h^{3}\left(1-v\right)}\zeta^{2}+\cdots =b\sigma R$$

Solving this equation and using the relationship $\varepsilon_{d}={\Omega \Lambda }_{d}b\zeta$, where, Ω is the conversion factor and $\Lambda_{d}$ is the dislocation density, the following relationship is obtained:

(6a) $$\varepsilon_{d}=\frac{1}{A_{2}^{d}}\sigma -\frac{1}{2}\frac{A_{3}^{d}}{{\left(A_{2}^{d}\right)}^{3}}\sigma^{2}+\cdots$$

with:

(6b) $$A_{2}^{d}=-\frac{G}{4\pi \Omega R\Lambda_{d}h^{2}\left(1-v\right)},\;\;A_{3}^{d}=\frac{G}{4\pi \Omega^{2}R\Lambda_{d}^{2}h^{3}\left(1-v\right)b}$$

Substituting Equations (3) and (6a,b) into Equation (2) yields:

(7) $$\varepsilon =\left(\frac{1}{A_{2}^{l}}+\frac{1}{A_{2}^{d}}\right)\sigma -\frac{1}{2}\left[\frac{A_{3}^{d}}{{\left(A_{2}^{d}\right)}^{3}}+\frac{A_{3}^{l}}{{\left(A_{2}^{l}\right)}^{3}}\right]\sigma^{2}+\cdots$$

The inverse expression can be written in the following form approximately:

(8) $$\sigma =A_{2}^{l}\varepsilon -\frac{1}{2}\left[A_{3}^{l}+\frac{A_{3}^{d}{\left(A_{2}^{l}\right)}^{3}}{{\left(A_{2}^{d}\right)}^{3}}\right]\varepsilon^{2}+\cdots$$

Substituting Equation (8) and Equation (6b) into the following wave motion equation:

(9) $$\rho \frac{\partial^{2}u}{\partial t^{2}}=\frac{\partial \sigma }{\partial x},\;\;\;\;\;\varepsilon =\frac{\partial u}{\partial x}$$

we have:

(10a) $$\frac{\partial^{2}u}{\partial t^{2}}-c^{2}\frac{\partial^{2}u}{\partial x^{2}}=c^{2}\left(\beta_{l}+\beta_{d}\right)\frac{\partial^{2}u}{\partial x^{2}}\frac{\partial u}{\partial x}+\cdots$$

with:

(10b) $$\beta_{l}=-\frac{A_{3}^{l}}{A_{2}^{l}},\;\;\;\;\;\;\;\beta_{d}=-\frac{A_{3}^{d}{\left(A_{2}^{l}\right)}^{2}}{{\left(A_{2}^{d}\right)}^{3}}=\frac{16\pi^{2}\Omega R^{2}\Lambda_{d}h^{3}{\left(1-v\right)}^{2}{\left(A_{2}^{l}\right)}^{2}}{G^{2}b}$$

where u is the total displacement, $\beta_{l},\;\;\beta_{d}$ are the nonlinear coefficients due to the material lattices and dislocations, respectively, $c=\sqrt{A_{2}^{l}/\rho }$ is the wave velocity, and ρ is the mass density of the material. Therefore, Equation (10a,b) is the nonlinear acoustic wave equation in materials with dislocations, from which the relationship between dislocation density and nonlinear harmonics can be established. The perturbation method is usually employed to solve this equation. 

Assuming the following solution form that is expanded with the Mach number:

(11) $$u=\sum_{i=1}^{n}M^{i}u_{i}.$$

where the Mach number $M=V_{0}/c$ and $V_{0}$ is the velocity amplitude of the vibration. Substituting Equation (11) into Equation (10a):

(12a) $$\frac{1}{c^{2}}\sum_{i=1}^{n}\frac{\partial^{2}}{\partial t^{2}}\left(M^{i}u_{i}\right)=\sum_{i=1}^{n}\frac{\partial^{2}}{\partial x^{2}}\left(M^{i}u_{i}\right)+\beta \sum_{i=1}^{n}\frac{\partial }{\partial x}\left(M^{i}u_{i}\right)\frac{\partial^{2}}{\partial x^{2}}\left(M^{i}u_{i}\right)+\cdots$$

with:

(12b) $$\beta =\beta_{l}+\beta_{d}$$

Factoring the above equation in terms of $M^{i}$,

(13a) $$M^{1}:\;\;\frac{1}{c^{2}}\frac{\partial^{2}u_{1}}{\partial t^{2}}=\frac{\partial^{2}u_{1}}{\partial x^{2}},$$

(13b) $$M^{2}:\;\frac{1}{c^{2}}\frac{\partial^{2}u_{2}}{\partial t^{2}}=\frac{\partial^{2}u_{2}}{\partial x^{2}}+\beta \frac{\partial u_{1}}{\partial x}\frac{\partial^{2}u_{1}}{\partial x^{2}},$$

(13c) $$M^{\cdots }:\;\cdots \cdots \;\;\;\;\;\;\;\;\;\;\;\;\;\;\;\;\;\;\;\;\;\;\;\;\;\;\;\;\;\;\;\;\;\;\;\;\;\;\;\;\;\;\;\;\;\;$$

from which the fundamental and higher harmonics of the propagating wave can be resolved by the perturbation method. 

The fundamental solution of Equation (13a) in the positive *x* direction has the form:

(14) $$u_{1}=A_{0}\cos\left(kx-\omega t\right),$$

where $A_{0}$ is the wave amplitude at the initial position (usually a driving source), k is the wave number, and ω is the angular frequency. 

For the second harmonic, substituting Equation (14) into Equation (13b),

(15) $$\frac{1}{c^{2}}\frac{\partial^{2}u_{2}}{\partial t^{2}}-\frac{\partial^{2}u_{2}}{\partial x^{2}}=\frac{1}{2}\beta A_{0}{}^{2}k^{3}\sin\left(2kx-2\omega t\right)$$

whose solution can be found to be,

(16) $$u_{2}=\frac{1}{8}\beta A_{0}{}^{2}k^{2}x\cos\left(2kx-2\omega t\right)$$

In the same way, the former order solutions are used to determine the next order one:

(17) $$\begin{array}{c} u_{3}=\beta^{2}[-\frac{1}{32}A_{0}{}^{3}k^{4}x^{2}\cos\left(kx-\omega t\right)-\frac{3}{32}A_{0}{}^{3}k^{3}x\sin\left(kx-\omega t\right)+\frac{1}{32}A_{0}{}^{3}k^{4}x^{2}\cos\left(3kx-3\omega t\right)\\ +\frac{5}{96}A_{0}{}^{3}k^{3}x\sin\left(3kx-3\omega t\right)] \end{array}$$

(18) $$\begin{array}{c} u_{4}=\beta^{3}[\left(-\frac{1}{96}A_{0}{}^{4}k^{6}x^{3}+\frac{1}{12}A_{0}{}^{4}k^{4}x\right)\cos\left(2kx-2\omega t\right)-\frac{33}{512}A_{0}{}^{4}k^{5}x^{2}\sin\left(2kx-2\omega t\right)\\ +\left(\frac{1}{96}A_{0}{}^{4}k^{6}x^{3}-\frac{11}{512}A_{0}{}^{4}k^{4}x\right)\cos\left(4kx-4\omega t\right)+\frac{5}{128}A_{0}{}^{4}k^{5}x^{2}\sin\left(4kx-4\omega t\right)] \end{array}$$

and so on, to obtain $u_{5},u_{6},\;\cdots \cdots ,u_{n}$. 

Substituting all the solutions into Equation (11) and combining the terms having the same harmonic frequency, we have,

(19) $$u=\sum_{n}A_{n}\sin\left(nkx-n\omega t+\arctan\frac{c_{n}}{s_{n}}\right)$$

where $A_{n}=\sqrt{s_{n}{}^{2}+c_{n}{}^{2}}$ and $\arctan\frac{c_{n}}{s_{n}}\;\left(\mathrm{for}\;n=1,\;2,\;3,\;4,\;\cdots \right)$ are the amplitude and phase of the nth harmonic wave component, respectively. $A_{n},s_{n},c_{n}$ are functions of propagation distance *x* and wave amplitude $A_{0}$ at the initial position. The first four orders are discussed in our work and are given as follows:

(20a) $$s_{1}=-\frac{3}{32}M^{3}\beta^{2}A_{0}{}^{3}k^{3}x,\;\;\;\;c_{1}=A_{0}M-\frac{1}{32}M^{3}\beta^{2}A_{0}{}^{3}k^{4}x^{2}$$

(20b) $$s_{2}=-M^{4}\beta^{3}\frac{33}{512}A_{0}{}^{4}k^{5}x^{2},\;\;\;\;c_{2}=\frac{1}{8}M^{2}\beta A_{0}{}^{2}k^{2}x+M^{4}\beta^{3}\left(-\frac{1}{96}A_{0}{}^{4}k^{6}x^{3}+\frac{1}{12}A_{0}{}^{4}k^{4}x\right)$$

(20c) $$s_{3}=\frac{5}{96}M^{3}\beta^{2}A_{0}{}^{3}k^{3}x,\;\;\;\;\;c_{3}=\frac{1}{32}M^{3}\beta^{2}A_{0}{}^{3}k^{4}x^{2}$$

(20d) $$s_{4}=\frac{5}{128}M^{4}\beta^{3}A_{0}{}^{4}k^{5}x^{2},\;\;\;\;\;\;c_{4}=M^{4}\beta^{3}\left(\frac{1}{96}A_{0}{}^{4}k^{6}x^{3}-\frac{11}{512}A_{0}{}^{4}k^{4}x\right)$$

(20e) $$M=\frac{V_{0}}{c}=\frac{\omega A_{0}}{c}$$

Through the above formulations, the relationship between the dislocation density and high order harmonics can be deduced. 

### 2.2. Nonlinearity Due to Micro-Cracks at Late Stage Fatigue

**Figure 2:**
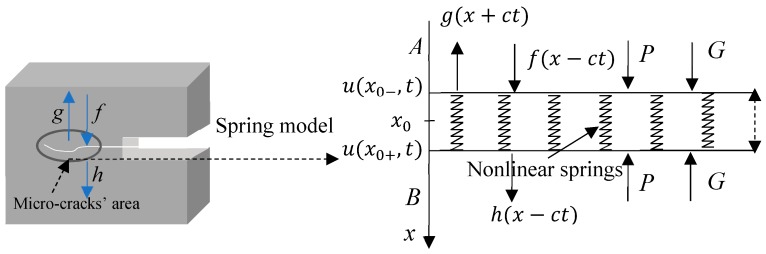
Nonlinear spring model.

While the fatigue of the metals is gradually strengthened, a further increase of the dislocation density leads to the occurrence of micro-cracks, which means a weak interface is produced in the material. The expansion of the micro-cracks will change the nonlinear behavior of the transmission wave, in both its transmitted energy and harmonics distribution. The acoustic nonlinearity mechanism in this case differs from that of the dislocation model. It can be established with the following nonlinear spring model, as shown in Figure 2.

*A* and *B* are two planes from either side of a crack interface. They are connected by the interfacial force, behaving like springs. Supposing that the displacement of an incident acoustic wave at plane *A* is f(x−ct)*,* and g(x+ct), h(x−ct) represents that of the reflected and transmitted wave, respectively. *P* is used to simulate the static force of the closed crack, and *G* is the joint dynamic force caused by the motion of the interface. The midpoint of the interface is taken as the original point “$x_{0}$”, where $x_{0-}$, $x_{0+}$ represent the upper side *A* and the lower side *B* of the interface. $u\left(x_{0-},t\right)$, $u\left(x_{0+},t\right)$ are the displacement of sides *A* and *B*, respectively. The relationship between the interface spacing $y\left(t\right)=u\left(x_{0+},t\right)-u\left(x_{0-},t\right)$ and G(y), F(t) is given as the following [16,17],

(21) $$\rho c\dot{y}\left(t\right)=2G\left(y\right)-2F\left(t\right)$$

where F(t) is caused by the incident ultrasonic wave,

(22) $$F\left(t\right)={\left(-\rho c^{2}\frac{\partial f\left(x-ct\right)}{\partial x}\right)}_{x=x_{0}}$$

From Equation (21), the variation of the crack pitch y(t) is related to both the joint dynamic force and ultrasonic force, as expressed in Equation (22). G(y) is usually nonlinear, whose relationship with interface spacing is given by the nonlinear spring model.

(23) $$G\left(y\right)=-K_{1}y+K_{2}y^{2}+\cdots$$

where $K_{1},\;K_{2}$ are the linear and nonlinear stiffness of the spring, respectively, which are related to the interface conditions. Considering the former dislocation model, the incident wave was already nonlinear because of the dislocation damage during the early stage before it propagated through the micro-cracks’ area, so it can be obtained from the former results at position $x_{0}$ in the following form according to Equation (19):

(24a) $$f\left(x-ct\right)=\underset{n}{\sum }A_{n}\sin\left(nkx_{0}-n\omega t+\varphi_{n}\right)$$

with:

(24b) $$\varphi_{n}=\arctan\frac{c_{n}}{s_{n}},n=1,2,3\cdots$$

Substitute Equations (23) and (24a,b) into Equation (21), then,

(25) $$\dot{y}+\frac{2K_{1}}{\rho c}y-\frac{2K_{2}}{\rho c}y^{2}-\cdots =2\underset{n}{\sum }n\omega A_{n}\cos\left(nkx_{0}-n\omega t+\varphi_{n}\right)$$

The solution of the above equation is composed of two parts: one linear part $y_{1}$ and a series of nonlinear parts $y_{2},\;\ldots$

(26) $$y=y_{1}+y_{2}+\cdots ,\;\;\;y_{1}\gg y_{2}\gg \cdots$$

Then,

(27) $$\dot{y_{1}}+\dot{y_{2}}+\frac{2K_{1}}{\rho c}\left(y_{1}+y_{2}+\cdots \right)-\frac{2K_{2}}{\rho c}{\left(y_{1}+y_{2}+\cdots \right)}^{2}-\cdots =2\underset{n}{\sum }n\omega A_{n}\cos\left(nkx_{0}-n\omega t+\varphi_{n}\right)$$

The linear part $y_{1}$ is easy to obtain by solving Equation (28):

(28) $$\dot{y_{1}}+\frac{2K_{1}}{\rho c}y_{1}=2\sum_{n}n\omega A_{n}\cos\left(nkx_{0}-n\omega t+\varphi_{n}\right)$$

Then,

(29) $$y_{1}=2\sum_{n}\frac{nA_{n}}{\sqrt{4K_{1}{}^{2}/n^{2}\omega^{2}\rho^{2}c^{2}+n^{2}}}\cos\left(nkx_{0}-n\omega t+\varphi_{n}+\phi_{n}\right)$$

where $\phi_{n}=\arctan\frac{n\omega \rho c}{2K_{1}}$, and $y_{2}$ can be resolved similarly from the following equation, while the small terms $y_{1}y_{2},\;\;y_{2}{}^{2},\ldots$ are omitted.

(30) $$\dot{y_{2}}+\frac{2K_{1}}{\rho c}y_{2}=\frac{2K_{2}}{\rho c}y_{1}{}^{2}$$

The harmonics of four orders are considered in the result,

(31) $$\begin{array}{c} y=\frac{2A_{1}\cos\left(kx_{0}-\omega t+\varphi_{1}+\phi_{1}\right)}{\sqrt{4K_{1}{}^{2}/\omega^{2}\rho^{2}c^{2}+1}}+\frac{4A_{2}\cos\left(2kx_{0}-2\omega t+\varphi_{2}+\phi_{2}\right)}{\sqrt{4K_{1}{}^{2}/\omega^{2}\rho^{2}c^{2}+4}}+\frac{6A_{3}\cos\left(3kx_{0}-3\omega t+\varphi_{3}+\phi_{3}\right)}{\sqrt{4K_{1}{}^{2}/9\omega^{2}\rho^{2}c^{2}+9}}+\\ \frac{8A_{4}\cos\left(4kx_{0}-4\omega t+\varphi_{4}+\phi_{4}\right)}{\sqrt{4K_{1}{}^{2}/16\omega^{2}\rho^{2}c^{2}+16}}-\frac{2A_{1}{}^{2}K_{2}\sin\left(2kx_{0}-2\omega t+2\varphi_{1}+2\phi_{1}+\phi_{2}\right)}{\left(4K_{1}{}^{2}/\omega^{2}\rho^{2}c^{2}+1\right)\sqrt{4K_{1}{}^{2}+4\omega^{2}\rho^{2}c^{2}}}-\frac{8K_{2}A_{2}{}^{2}\sin\left(4kx_{0}-4\omega t+\varphi_{4}+2\phi_{2}+\phi_{4}\right)}{\left(4K_{1}{}^{2}/\omega^{2}\rho^{2}c^{2}+4\right)\sqrt{4K_{1}{}^{2}+16\omega^{2}\rho^{2}c^{2}}}+\\ \frac{4K_{2}A_{2}A_{1}}{\sqrt{\left(4K_{1}{}^{2}/\omega^{2}\rho^{2}c^{2}+1\right)\left(4K_{1}{}^{2}/\omega^{2}\rho^{2}c^{2}+4\right)}}\left(\frac{\cos\left(kx_{0}-\omega t+\varphi_{3}-\varphi_{1}+\phi_{2}\right)}{\sqrt{4K_{1}{}^{2}+\omega^{2}\rho^{2}c^{2}}}+\frac{\cos\left(3kx_{0}-3\omega t+\varphi_{3}+\varphi_{1}+\phi_{1}+\phi_{2}+\phi_{3}\right)}{\sqrt{4K_{1}{}^{2}+9\omega^{2}\rho^{2}c^{2}}}\right)+\cdots \end{array}$$

The transmitted wave through the spring interface can be obtained with the following equations [16,17],

(32) $$h\left(x-ct\right)=f\left(x-ct\right)+\frac{1}{2}y\left(t-\frac{x}{c}\right)$$

Then,

(33) $$\begin{array}{c} h\left(x-ct\right)=A_{1}\sin\left(kx_{0}-\omega t+kx+\varphi_{1}\right)+A_{2}\sin\left(2kx_{0}-2\omega t+2kx+\varphi_{2}\right)+A_{3}\sin(3kx_{0}-3\omega t+\\ 3kx+\varphi_{3})+A_{4}\sin\left(4kx_{0}-4\omega t+4kx+\varphi_{4}\right)+\frac{A_{1}\cos\left(kx_{0}-\omega t+kx+\varphi_{1}+\phi_{1}\right)}{\sqrt{4K_{1}{}^{2}/\omega^{2}\rho^{2}c^{2}+1}}+\\ \frac{2A_{2}\cos\left(2kx_{0}-2\omega t+2kx+\varphi_{2}+\phi_{2}\right)}{\sqrt{4K_{1}{}^{2}/4\omega^{2}\rho^{2}c^{2}+4}}+\frac{3A_{3}\cos\left(3kx_{0}-3\omega t+3kx+\varphi_{3}+\phi_{3}\right)}{\sqrt{4K_{1}{}^{2}/9\omega^{2}\rho^{2}c^{2}+9}}+\frac{4A_{4}\cos\left(4kx_{0}-4\omega t+4kx+\varphi_{4}+\phi_{4}\right)}{\sqrt{4K_{1}{}^{2}/16\omega^{2}\rho^{2}c^{2}+16}}-\\ \frac{2A_{1}{}^{2}K_{2}\sin\left(2kx_{0}-2\omega t+2kx+2\varphi_{1}+2\phi_{1}+\phi_{2}\right)}{\left(4K_{1}{}^{2}/\omega^{2}\rho^{2}c^{2}+1\right)\sqrt{4K_{1}{}^{2}+4\omega^{2}\rho^{2}c^{2}}}-\frac{4K_{2}A_{2}{}^{2}\sin\left(4kx_{0}-4\omega t+4kx+2\varphi_{2}+2\phi_{2}+\phi_{4}\right)}{\left(4K_{1}{}^{2}/\omega^{2}\rho^{2}c^{2}+4\right)\sqrt{4K_{1}{}^{2}+16\omega^{2}\rho^{2}c^{2}}}+\\ \frac{2K_{2}A_{2}A_{1}}{\sqrt{\left(4K_{1}{}^{2}/\omega^{2}\rho^{2}c^{2}+1\right)\left(4K_{1}{}^{2}/\omega^{2}\rho^{2}c^{2}+4\right)}}\left(\frac{\cos\left(kx_{0}-\omega t+kx+\varphi_{3}-\varphi_{1}+\phi_{2}\right)}{\sqrt{4K_{1}{}^{2}+\omega^{2}\rho^{2}c^{2}}}+\frac{\cos\left(3kx_{0}-3\omega t+3kx+\varphi_{3}+\varphi_{1}+\phi_{1}+\phi_{2}+\phi_{3}\right)}{\sqrt{4K_{1}{}^{2}+9\omega^{2}\rho^{2}c^{2}}}\right)+\cdots \end{array}$$

The transmitted coefficient of each order harmonic can be obtained with the above solution,

(34) $$T_{n}=\sqrt{\frac{4K_{1}{}^{2}/n^{2}\omega^{2}\rho^{2}c^{2}+2n^{2}+2n\sin\phi_{n}\sqrt{4K_{1}{}^{2}/n^{2}\omega^{2}\rho^{2}c^{2}+n^{2}}}{4K_{1}{}^{2}/n^{2}\omega^{2}\rho^{2}c^{2}+n^{2}}},\;n=1,2,3,4\ldots$$

### 2.3. Computation Results

**Figure 3:**
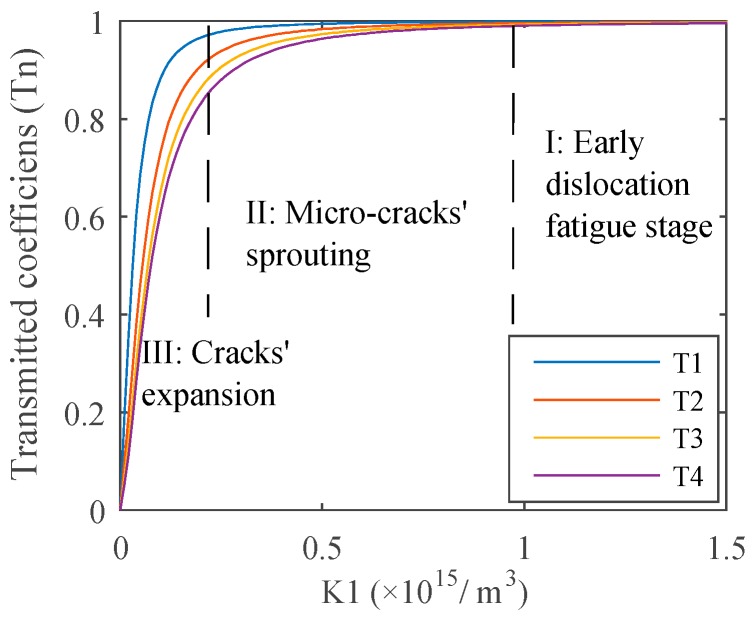
Relationship between the transmitted coefficient of each order harmonic (the fundamental wave T_1_, the second-order harmonics T_2_, the third-order harmonics T_3_, the fourth-order harmonics T_4_) and spring stiffness.

The transmission coefficients are related to the stiffness $K_{1}$, as shown in Figure 3, the second-order terms are small enough to be ignored compared with the linear parts. The computation parameters of the aluminum alloy are chosen as an example, and the values of relevant parameters are referenced from the work of John H. Cantrell [9], which are given in Table 1. 

**Figure 4:**
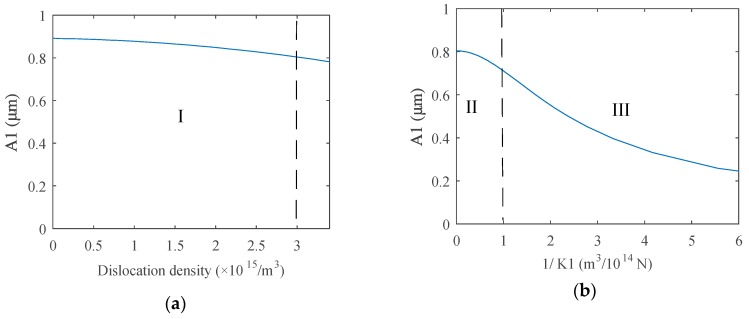

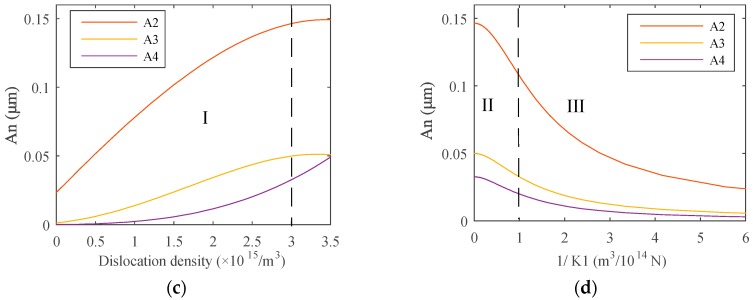
Amplitude variation of (**a**) the fundamental wave A_1_, (**b**) the second-order harmonics A_2_, (**c**) the third-order harmonics A_3_, and (**d**) the fourth-order harmonics A_4_ during the fatigue process, which is expressed by dislocation density for early stage and spring stiffness for the later stage.

It is obvious that the transmitted coefficient of each order harmonic approaches one, while $K_{1}\to \infty$, usually $K_{1}\geq 1\times 10^{15}\;N/m^{3}$, which can be considered as full transmission, as shown in Region I: the early dislocation fatigue stage in Figure 3. Therefore, during this stage, acoustic nonlinearity mainly results from dislocations and wave energy transmitted completely without the effects of the weak interface, as described in the dislocation dipole model. Once the micro-cracks sprout, from $K_{1}=1\times 10^{15}\;N/m^{3}$, spring stiffness begins to decline, and the energy of the transmitted wave drops gradually, as shown in Region II: micro-crack sprouting stage. A large amount of micro-crack sprouting leads to the rapid expansion of the crack length, which reduces the transmitted wave energy clearly; it is the last stage of the fatigue process in Region III: crack expansion stage. During the micro-crack sprouting stage, nonlinearity increased, owing to the increasing dislocation density being very weak compared to the effects of spring the stiffness decline with continuous fatigue loading. Relative results are given in Figure 4. Computation parameters are also given in Table 1. The initial amplitude of each order harmonic is chosen from the above dislocation model results at $x_{0}=50\mathrm{mm},\;\;\mathrm{driving}\;\mathrm{source}\;\mathrm{amplitude}\;A_{0}=1\mu m$. Assuming that micro-cracks sprout while the dislocation density is about $3\times 10^{15}\;m^{-3}$, which is the early fatigue stage, the harmonics variations are given in Figure 4a,c. After this stage, the spring stiffness in the computation model was taken from ∞→0, which means 1/$K_{1}$ from 0→∞, as shown in Figure 4b,d; the dislocation density was assumed as constant during this process.

The above results show that acoustic nonlinearity is very weak for materials without damages, as given in Figure 4c, while the dislocation density is zero. However, with the increase of dislocation density, which means the deepening of fatigue damage, acoustic nonlinearity becomes much heavier. The fundamental wave amplitude is going to decrease, while harmonic amplitudes begin to rise due to the energy distribution law that energy transfers from the lower order signals to the higher orders, as shown in Region I in Figure 4a,c. Then, acoustic nonlinearity indicates the fatigue degree in materials during the early fatigue stage. Micro-cracks sprout with further fatigue loading, and the value of spring stiffness begins to drop, whose effects on each order harmonic are much heavier than that of the increasing dislocation density, so the harmonic amplitudes are going to decrease, as shown in Region II in Figure 4b,d. The energy of each order transmitted wave declines very quickly with the decreasing stiffness, owing to the rapid expansion of cracks in Region III. 

## 3. Experiment Results and Discussion

**Figure 5:**
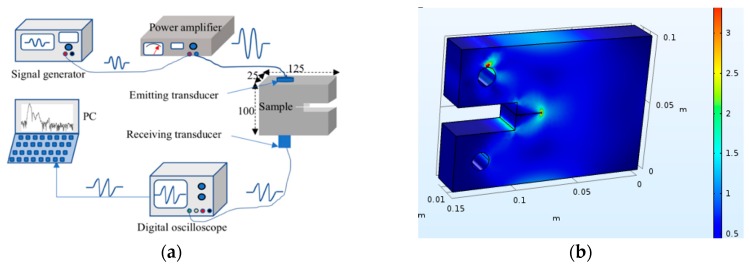
(**a**) The experiment system. (**b**) The stress distribution during the fatigue test.

Based on the above analysis for the theoretical models during the fatigue process, the following experiments were designed and carried out. The experimental setup employed is represented in Figure 5a. It was composed of: a signal generator (Tektronix AFG1022, Tektronix, Shanghai, China), a power amplifier (Model 2100L RF Power Amplifier, ENI, Rochester, NY, USA), two transducers (central frequency of 1.1 MHz for the emitting one and 2.3 MHz for the receiving one, respectively), and a digital oscilloscope (Agilent Technologies MSO7032A, Agilent Technologies Inc., Santa Clara, CA, USA).

The receiving transducer sensitivity at 1.1 MHz was about −11.4 dB, for a signal of 2.2 MHz (the second-order signal), 3.3 MHz (the third-order one), and 4.4 MHz (the fourth-order one), and the sensitivities were −2 dB, −6 dB, and −12 dB, respectively. The gains of the receiving transducers were compensated in the following high-order harmonic amplitude measurements.

**Figure 6:**
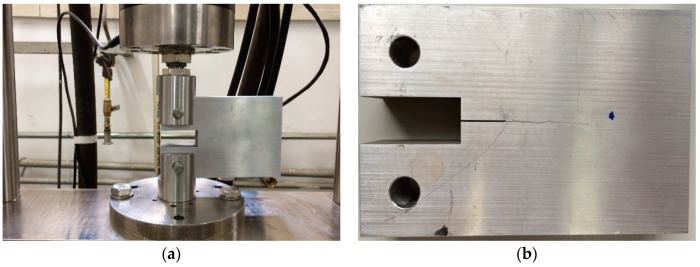
(**a**) The fatigue test. (**b**) The fatigue sample with a crack.

Before the preparation of the fatigue specimen, the fatigue model of the aluminum alloy specimen was established using finite element software (COMSOL Multiphysics 5.3, COMSOL Inc., Stockholm, Sweden). As in the fatigue test, the same size model was established with the same material. The lower surface boundary was fixed, and the upper surface boundary was loaded with the maximum stress during the fatigue test. The total stress distribution of this specimen in the fatigue process was analyzed, and the results are given in Figure 5b. It was shown that the stress in the test piece was concentrated along the initial grooving. If the fatigue degree increased constantly, tiny cracks would start to sprout and gradually extend forward. According to the calculation results of the model, the fatigue test was carried out on the sample by selecting appropriate parameters, the process of which is shown in Figure 6a.

**Figure 7:**
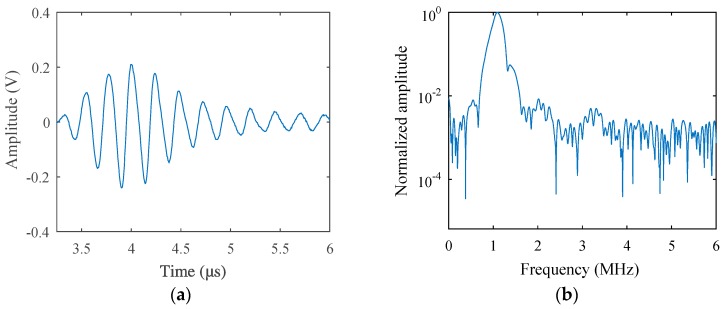
(**a**) Transmitted signals received from the fatigue sample (Loading Cycle 0) in the time-domain and (**b**) in the frequency-domain (excitation voltage 200 V).

**Figure 8:**
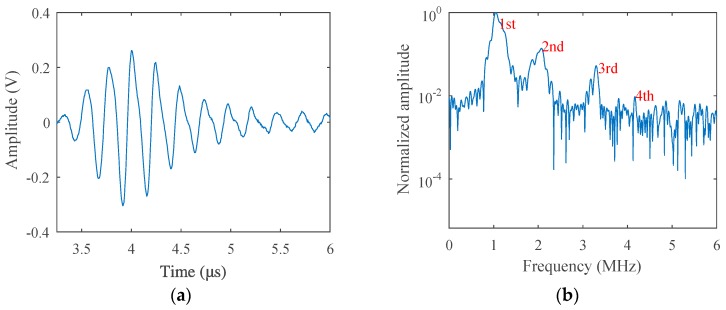
(**a**) Transmitted signals received from the fatigue sample (Loading Cycle 5000) in the time-domain and (**b**) in the frequency-domain (excitation voltage 200 V).

**Figure 9:**
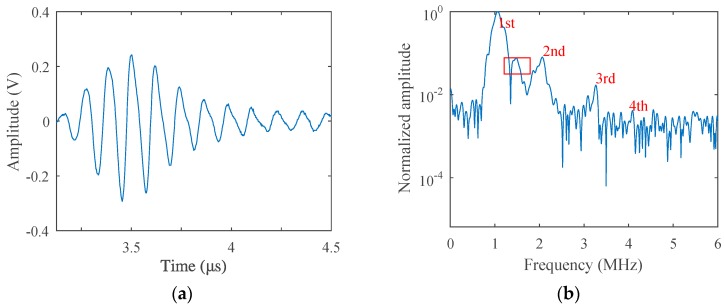
(**a**) Transmitted signals received from the fatigue sample (Loading Cycle 20,000) in the time-domain and (**b**) in the frequency-domain (excitation voltage 200 V).

**Figure 10:**
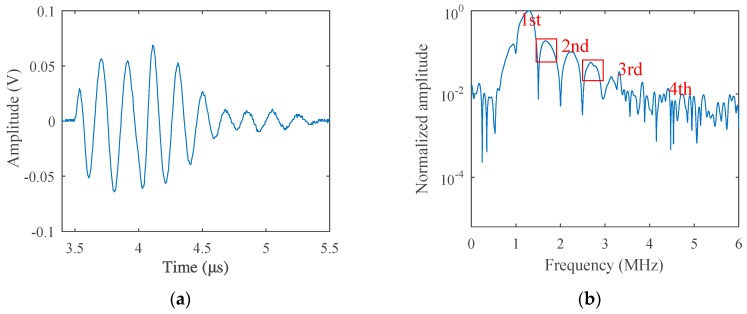
(**a**) Transmitted signals received from the fatigue sample (Loading Cycle 29800) in the time-domain and (**b**) in the frequency-domain (excitation voltage 200 V).

The fatigue test was carried out at room temperature using the stress-loaded control mode, during which the average fatigue loading force was 5 kN and the loading frequency was 5 Hz. After a certain period of loading, nonlinear acoustic measurements were carried out and then redetected after another period of loading, until micro-cracks sprouted in the sample. The loading cycle was 500, 1500, 3000, 5000, 10,000, 15,000, 20,000, 25,000, and 29,800, respectively. Before the fatigue loading, acoustic measurement was performed to get the result of the non-damaged sample as a comparison, which is given in Figure 7. While the loading cycle was about 5000 during the fatigue test, acoustic nonlinearity increased very clearly in the received signals, as shown in Figure 8. Micro-cracks were observed from the end of the preset grooving after 20,000 cycles of loading, the results of which are given in Figure 9. Once micro-cracks sprouted, continuous loading accelerated their expansion. Fatigue loading was performed until the crack could be seen clearly in the sample, as shown in Figure 6b, whose loading cycle was about 29,800, and the measurement results are given in Figure 10. 

Comparing the results in Figure 7a and Figure 8a, the signal received from the sample after the fatigue test (Loading Cycle 5000) deformed much more heavily than that of the non-fatigue situation (Loading Cycle 0), which is much more obvious in the spectrum results in Figure 7b and Figure 8b. After fatigue loading, which means the increase of dislocation density, as explained in the dislocation dipole model, harmonics caused by the damage increased also; therefore, the spectrum was much richer in harmonic components, and each-order harmonic could be seen more clearly than the result of the non-damage situation. 

The difference is not obvious in the time-domain result in Figure 9a, except that the transmitted energy is a little lower compared with the result in Figure 8a. However, in the spectrum results in Figure 9b, not only the harmonic amplitudes are a little lower, but another frequency component whose frequency is between the fundamental wave and second-order harmonics appears, compared with the results in Figure 8b, which may be caused by the wave scattering of the crack tip or the spatial variation in the stiffness of the springs. These results are much more apparent in Figure 10. As micro-cracks expanded very quickly with continuous loading, the energy of the transmitted wave in this stage was much lower than that in the former situations, as given in the time-domain result, which was consistent with the spring model result. In addition, more spectrum components appeared in the spectrum result, as marked with rectangles in Figure 10b. Because the crack surface was rough and curved, this may have led to more scattering waves and generated more other intermediate frequency components in the result. It is very complicated to describe in theoretical models and will be studied in our next work.

**Figure 11:**
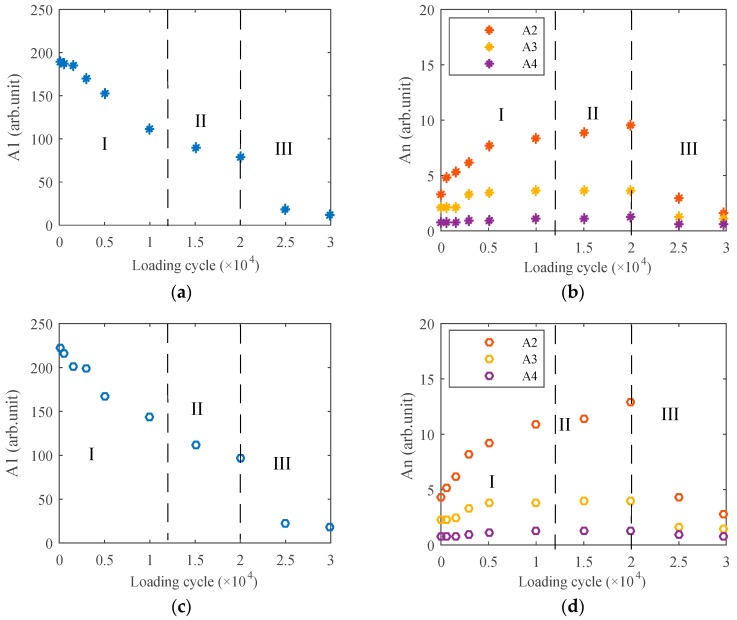

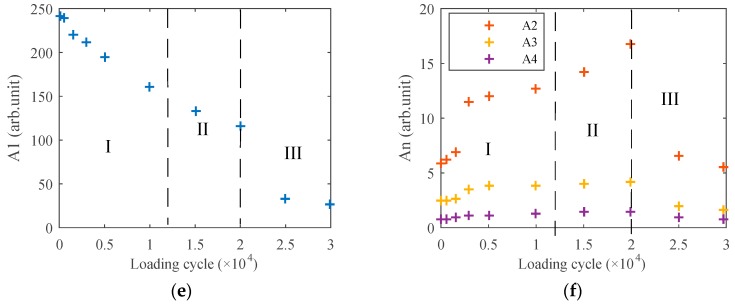
Amplitude variation of (**a**) the fundamental wave A_1_, (**b**) the high order harmonics A_n_ (excitation voltage 150 V), (**c**) the fundamental wave A_1_, (**d**) the high order harmonics A_n_ (excitation voltage 175 V), (**e**) the fundamental wave A_1_, and (**f**) the high order harmonics A_n_ (excitation voltage 200 V) during the whole fatigue process.

The above results are some typical measurements for explaining the acoustic nonlinearity variation during different fatigue stages. Detailed amplitude variation of each order harmonic with fatigue degree, which is described by the fatigue loading cycle, is shown in Figure 11. During each measurement, excitation voltages were taken as 150 V (Figure 11a,b), 175 V (Figure 11c,d), and 200 V (Figure 11e,f), respectively.

Generally, the experiment results can also be divided into three stages: (I) the early dislocation fatigue stage, (II) the micro-crack sprouting stage, and (III) the crack expansion stage, which is consistent with the theoretical model results, as shown in the above figures. While the loading cycle was about less than 10,000, harmonic components increased obviously with continuous loading, which can be treated as the early stage of fatigue. In this stage, acoustic nonlinearity mainly came from dislocations, and it can be used to assess the early stage fatigue degree, as explained in the theoretical model of Region I. Further loading led to micro-cracks sprouting inside of the metals, and the occurrence of a weak interface reduced the transmitted wave energy. Therefore, the increasing trend of the harmonics amplitude became slow even with higher dislocation density, which was the transition stage. The boundary line of this stage was between 10,000 and 15,000 cycles, approximately, which corresponds to the middle part in Figure 4. While the loading cycle was about 20,000, cracks were seen clearly at the end of the preset grooving, which meant the beginning of the crack expansion stage. The amplitude of each order harmonic declined quickly during this stage, the results of which are consistent with the theoretical results of Region III in Figure 4.

## 4. Conclusions

Acoustic nonlinearity during the fatigue process in metals can be divided into three stages: (I) the early dislocation fatigue stage, (II) the micro-crack sprouting stage, and (III) the crack expansion stage, which can be described by nonlinear spring models combined with dislocation theory. Varying spring stiffness is chosen to connect different stages in the fatigue process. For the early dislocation fatigue stage, spring stiffness approaches infinity, and acoustic nonlinearity mainly results from dislocations, the results of which can be used for early fatigue evaluation. Once micro-cracks sprout in Region II, spring stiffness will drop, which reduces the transmitted wave energy and makes the harmonics fall. The decreasing trends become steep quickly, owing to the crack’s expansion, and additional spectrum components are generated in the results with crack occurrence in Region III. These may be the effects of the scattering wave on rough crack interface or the spatial variation in the stiffness of the springs. This phenomenon is complicated, and studying its nonlinear acoustic mechanism is our next work.

## Figures and Tables

**Table 1:** Computational parameters of the aluminum alloy.

Parameters	Value	Unit
$A_{2}^{l}$	109	GPa
$A_{3}^{l}$	−510	GPa
G	28.6	GPa
*b*	0.4	mm
Ω	0.33	/
*R*	0.33	/
h	5	nm
v	0.33	/
*c*	6250	m/s
ρ	2700	kg/m^3^
*f*	1	MHz
